# Bilateral interdental cleaning after powered toothbrushing: an in vitro comparison of interdental brushes and elastomeric interdental picks

**DOI:** 10.1186/s12903-026-08946-8

**Published:** 2026-06-18

**Authors:** Sabrina Naeve, Miriam Cyris, Ann-Kristin Härdter, Martin Straßburger, Thomas Rinder, Christof E. Dörfer, Sonja Sälzer, Christian Graetz

**Affiliations:** 1 Clinic of Conservative Dentistry and Periodontology, University Hospital Schleswig-Holstein, Kiel, 24105 Germany; 2 Institute of Mechatronics, Computer Science and Electrical Engineering, Kiel University of Applied Sciences, Kiel, Germany; 3 Dental Practices Poststraße, Poststraße 17, Hamburg, 20354 Germany

**Keywords:** Oral hygiene, Interdental brushes, Mechanical plaque control, Interdental cleaning efficacy

## Abstract

**Background:**

Plaque removal in interdental areas (IDR) remains limited when using powered toothbrushing (PT) alone. Although adjunctive interdental cleaning is widely recommended, quantitative data on the incremental benefit of bilateral interdental application are limited. This in vitro study evaluated cleaning potential following sequential interdental cleaning after PT and compared the bilateral incremental effect between interdental brushes (IDB) and elastomeric interdental picks (IDP).

**Methods:**

A validated in vitro model (verified for reproducibility and standardised quantitative ECE measurement under controlled in vitro conditions) using a standardised artificial biofilm model to quantify interdental experimental cleaning efficacy (ECE, %). Replicated IDRs of different morphologies and sizes simulating open posterior IDR with reduced papillary support were cleaned sequentially using PT alone, PT followed by one-sided interdental application, and PT followed by bilateral interdental application. The primary outcome was the total incremental cleaning effect after PT (ΔECE2). The additional benefit of second-side application (ΔECE3) was analysed as endpoint. Data were analysed using non-parametric tests with Bonferroni correction.

**Results:**

PT alone resulted in low interdental cleaning efficacy (IDB/ IDP series: 7.5 ± 2.7%/ 10.2 ± 4.1%; *p* < 0.001). Following bilateral interdental cleaning, overall ECE increased to 81.2 ± 18.3% for IDB (IDP: 54.7 ± 17.6%; *p* < 0.001). The total incremental effect after PT was significantly greater for IDB (ΔECE2 73.7 ± 17.8%) than for IDP (44.5 ± 20.6%; *p* < 0.001). In contrast, the additional benefit of second-side application was greater for IDP vs. IDB (ΔECE3: 21.4 ± 14.1% vs. 9.5 ± 12.2%; *p* < 0.001). The superiority of IDB in total cleaning efficacy was consistent across IDR morphologies and sizes.

**Conclusions:**

Within the limitations of this in vitro study, PT alone was insufficient for effective cleaning of simulated open posterior interdental areas with reduced papillary support. Cylindric IDBs demonstrated superior overall performance across all tested conditions, while conical IDPs derived a comparatively greater relative benefit from bilateral access.

**Supplementary Information:**

The online version contains supplementary material available at 10.1186/s12903-026-08946-8.

## Background

Effective plaque control in interdental areas (IDR) remains a major challenge in daily oral hygiene [1]. While powered toothbrushes (PT) have been shown to improve plaque removal on accessible tooth surfaces [2], their efficacy in open IDRs is limited due to anatomical constraints [3]. Consequently, adjunctive interdental cleaning devices are recommended to enhance biofilm removal in these areas. Interdental brushes (IDBs) with a metal core are widely regarded as the most effective mechanical approach for plaque removal in open IDR [1, 4]. Metal-free interdental rubber picks (IDPs) have been introduced as an alternative, offering improved comfort and ease of use, which may increase patient compliance [5]. However, previous investigations using validated and reproducible experimental models demonstrated that cleaning efficacy varies substantially depending on the type and size of the interdental device, as well as on interdental morphology and gap dimension [3, 6, 7]. In daily practice, interdental cleaning is influenced by access, ease of use, and individual patient-related factors, as highlighted in recent consensus recommendations by Thomassen, Slot [1]. These practical considerations may result in application from a single (buccal) aspect. Whether bilateral interdental application provides a measurable additional benefit compared with unilateral use remains insufficiently quantified under standardized experimental conditions. Using our established in vitro setup for quantifying experimental cleaning efficacy (ECE) [6], the present study aimed to evaluate the in vitro cleaning potential of sequential application of a PT (GUM^®^ Playbrush; Sunstar Europe, Etoy, Switzerland) followed by interdental cleaning using IDBs (GUM^®^ TRAV-LER^®^) or IDP (GUM^®^ Soft-Picks^®^ Pro; Sunstar Suisse SA). The primary objective of this in vitro study was to investigate the additional in vitro cleaning benefit achieved by bilateral (buccal and oral) interdental application following PT and to compare this effect between IDB and IDP. We hypothesized that bilateral application would result in a further increase in interdental cleaning efficacy beyond one-sided use and that IDP would exhibit a greater additional benefit from two-sided application than IDB.

## Methods

### Test products

**Fig. 1:**
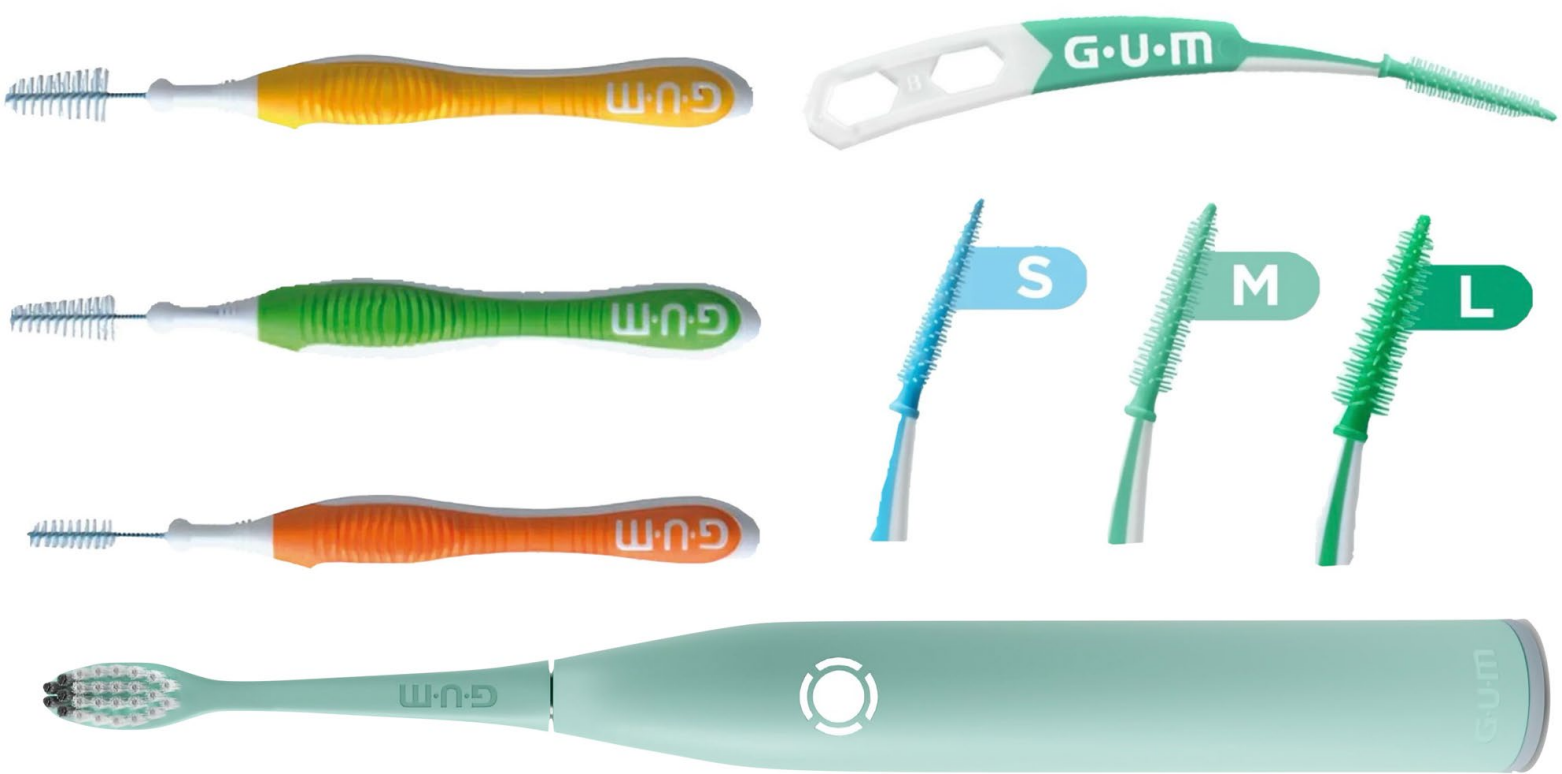
Test products used: GUM TRAV-LER in ISO sizes 2, 3 and 4 (IDB, left side in the picture); GUM Soft-Picks Pro in sizes S, M and L (IDP, right side in the picture) and GUM^®^ SMART ONE powered toothbrush (PT, below in the picture). Images edited by https://professional.sunstargum.com

In this in vitro study, conical IDP (GUM SOFT-PICKS^®^ Pro, Sunstar Suisse SA, Etoy, Switzerland) in small, medium and large sizes and IDB with a wire core and nylon filaments (diameters 0.8, 0.9, 1.2 mm; ISO 2–4; GUM^®^ TRAV-LER^®^, Sunstar Suisse SA, Etoy, Switzerland) were used (Fig. 1). The respective device size was selected according to predefined IDR replica dimensions using standardized fitting criteria derived from our previously validated experimental model [3, 6], which was verified for reproducibility and standardised quantitative ECE measurement under controlled in vitro conditions. This structured size-matching protocol was applied consistently across all specimens and was not based on subjective adjustment. The approach was intended to simulate clinically appropriate, anatomically adapted device selection.

### Experimental setup and image analysis

As explained in detail in our previous publication [6], we used a computer software (Autodesk Fusion 360, Autodesk Direct Limited, Hampshire, United Kingdom) and in vivo data of interdental morphologies [7–9] to design and print 3D composite replicas in stereolithography manner (Form 2, Formlabs Sommerville, MA, USA) by using liquid photopolymer resin (White Resin V04 (RS-F2-GPWH-04), Formlabs, Sommerville, MA, USA). The experimental setup was developed to ensure high reproducibility and dimensional accuracy [10].

**Fig. 2:**
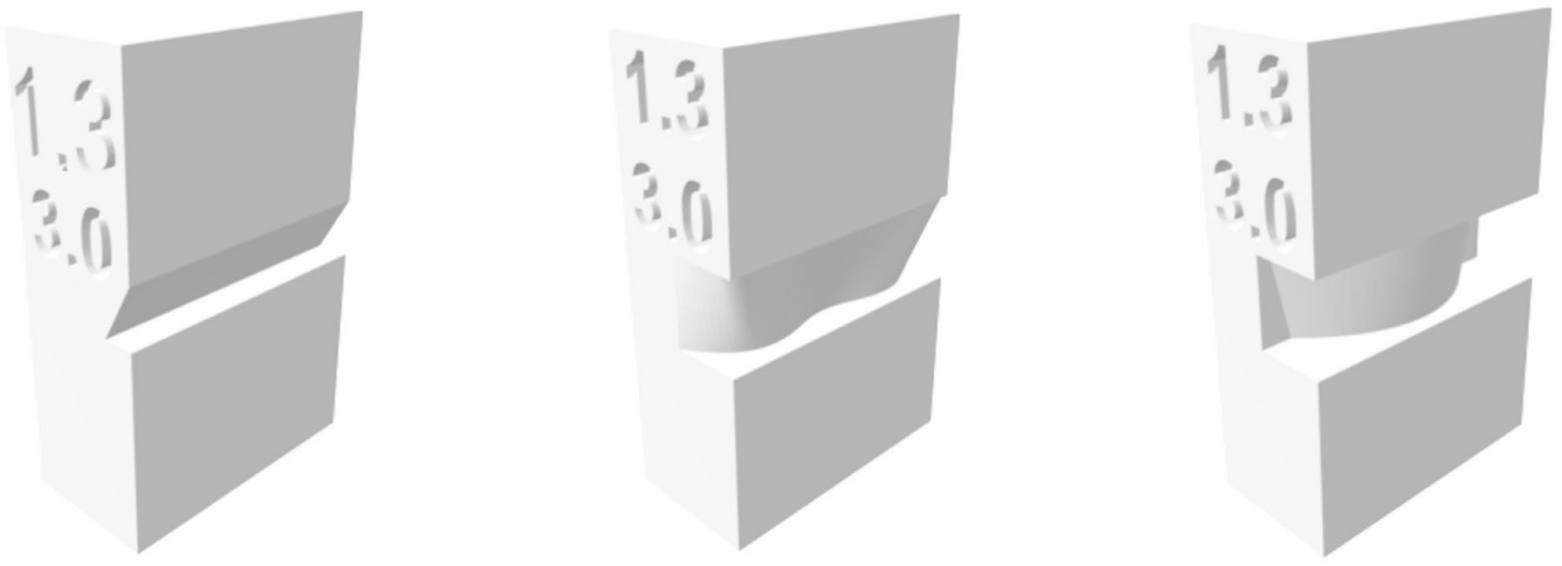
Illustration of the tested interdental area (IDR) morphology (from left to right: isosceles triangular, concave, convex) with 1.3 mm size (3,0 mm height) and all with 10 mm depth

We used three interdental IDR sizes of 1.0 mm (small), 1.1 mm (medium) and 1.3 mm (large) in four morphologies (isosceles triangle, convex, concave space, all of 3 mm height), all with 10 mm depth, resulting in nine different artificial interdental areas (Fig. 2). These IDRs simulating open posterior interdental sites with reduced papillary support.

**Fig. 3:**
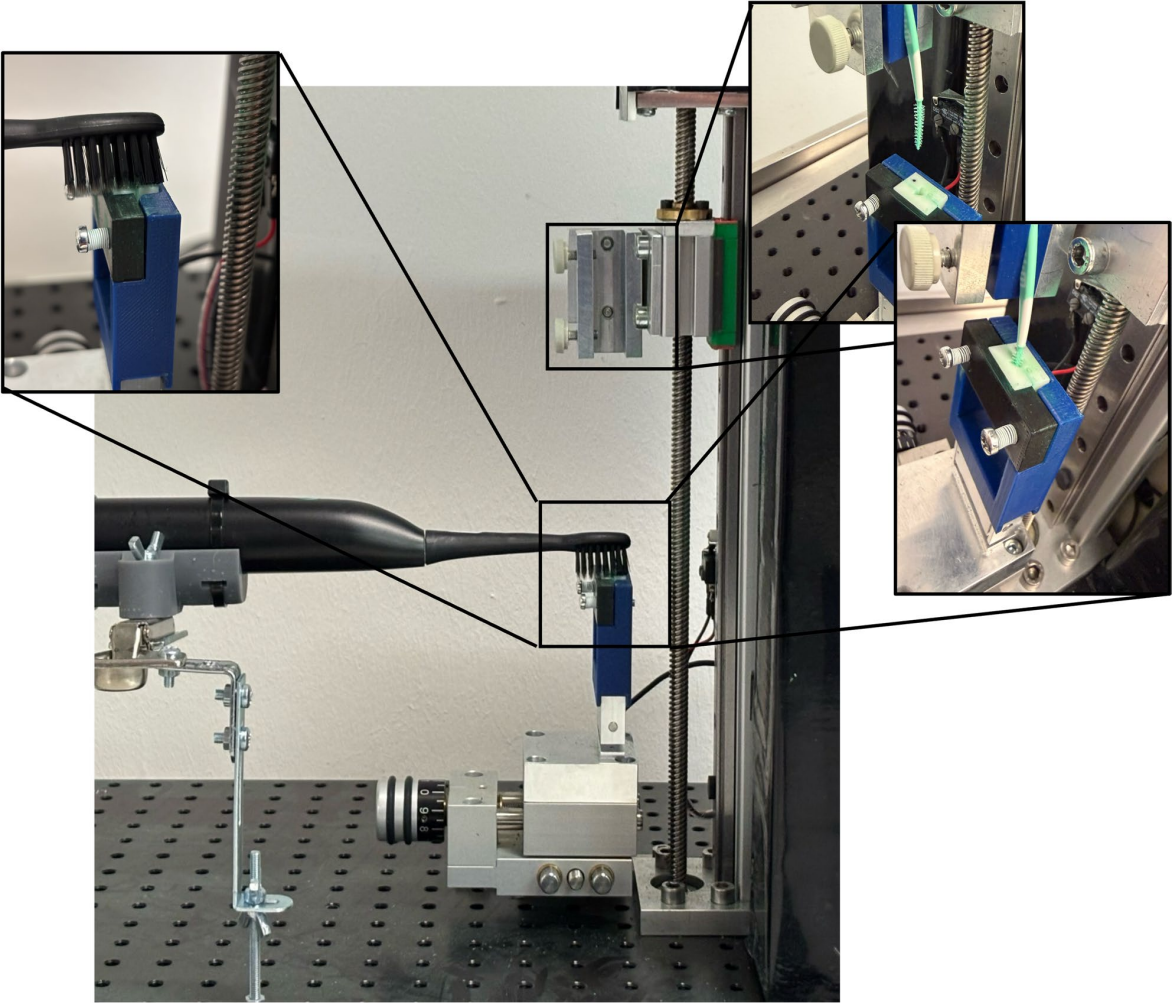
Experimental setup illustrating the simulation model with integrated sonic powered toothbrush (PT) and vertically aligned linear movement unit for standardized interdental device insertion. The toothbrush head was positioned at a 45° angle relative to the artificial interdental area (IDR) replica to simulate clinically recommended brushing technique. Insets demonstrate the fixation of the IDR replica and the controlled insertion of an interdental pick (IDP) before and during introduction into the IDR replica

Subsequently, the IDR replicas were stained by one investigator (S.N.) with Occlu Spray Plus (Hager & Werke, Duisburg, Germany) as described in previous studies [6, 11, 12]. It was used as a standardised plaque surrogate, while this agent allows reproducible surface staining and quantification of cleaned areas, it does not replicate the adhesion properties, structural complexity, or biomechanical characteristics of mature in vivo interdental biofilm. A standardized powder thickness (mean ± SD: 20 ± 5 μm) was ensured by a standardized procedure and appropriate time protocol. The baseline surface was digitally photographed (Canon EOS 400D Digital, Uxbridge, United Kingdom) and documented. Subsequently, powered toothbrushing was performed for 10 s using the test PT mounted in a fixed holder to ensure standardized positioning (Fig. 3). The brush head was aligned at a 45° angle relative to the IDR replica, following the principles of the Bass technique [13]. A constant contact force of 1.0 ± 0.3 N was applied and maintained via a calibrated spring mechanism, corresponding to clinically reported brushing forces for sonic toothbrushes [14]. No additional manual movement was performed. After completion of the PT phase, photographic documentation was conducted. Interdental cleaning devices (IDB or IDP) were then introduced into the IDR replica using the mechanical device ensuring standardized linear insertion at controlled speed (Fig. 3). Each IDB or IDP was moved ten times (10 in–out cycles) per application side. After one-sided application, the IDR replica was photographed (Supplemental Figure S1), and the procedure was repeated from the opposite side, followed by final photographic documentation. All images were captured under standardized conditions and were subsequently analysed using digital image subtraction. The images were extracted via XN View to enhance the most important area of the interdental space. Afterwards the picture section was edited by Photoshop in the same procedure as described in detail in our previous study [6] to get a digital image subtraction (ImageJ, NIH, Bethesda, MD, USA). Cleaned and non-cleaned areas were differentiated based on colour contrast and quantified using digital image subtraction. The ECE was calculated as the percentage reduction of the simulated biofilm area between the respective pre- and post-intervention images. Incremental cleaning effects (ΔECE) were derived from the sequential measurements to quantify the additional benefit of one-sided and two-sided interdental application. The reproducibility and validity of the experimental setup have been previously demonstrated [6].

### Sample size

The sample size was determined a priori based on our previously published and validated in vitro model, in which *n* = 25 samples per group were sufficient to detect a 5% difference in ECE with 80% statistical power [3, 6]. In the present factorial design including predefined IDR morphologies and sizes, equal numbers of replicates were allocated to each morphology–size–device combination (*n* = 10 per morphology–size–device configuration), resulting in *n* = 30 observations per device within each morphology and *n* = 30 observations per device within each size category. Stratified analyses by IDR morphology and size were pre-specified secondary analyses and were not individually powered to detect small subgroup differences. The overall allocation ensured adequate precision and statistical power for the primary comparison of total incremental cleaning efficacy (ΔECE2) between device types. The additional benefit of second-side application (ΔECE3) was analysed as a predefined mechanistic endpoint.

### Statistical analysis

All statistical analyses were performed using SPSS Statistics (Version 27, IBM Corp., Armonk, NY, USA). ECE is reported as mean ± standard deviation (SD) and median with interquartile range (IQR). Normality was assessed using the Kolmogorov–Smirnov test with Lilliefors correction (*p* < 0.001) and the Shapiro–Wilk test (*p* < 0.001). As data were not normally distributed, non-parametric statistical tests were applied. It should be noted, as device sizes of IDB/ IDP were selected according to interdental area dimensions to ensure size-fitted application, statistical analyses were conducted at device level (IDB vs. IDP) rather than at individual product size level. Between-group comparisons (IDB vs. IDP) for absolute ECE values at each application modality (PT alone, PT + one-sided, PT + two-sided) were performed using the two-sided Mann–Whitney U test. Bonferroni correction was applied for multiple testing across the three between-group comparisons. Within-group comparisons across sequential application modalities (PT vs. PT + one-sided; PT vs. PT + two-sided; PT + one-sided vs. PT + two-sided) were analysed using the paired Wilcoxon signed-rank test, as measurements were obtained sequentially on the same experimental replica. Bonferroni correction was applied separately within each device group across the three paired comparisons. Incremental cleaning efficacy was analysed using three predefined parameters: (1) ΔECE1 = ECE(PT + one-sided) − ECE(PT alone); (2) ΔECE2 = ECE(PT + two-sided) − ECE(PT alone) and (3) ΔECE3 = ECE(PT + two-sided) − ECE(PT + one-sided). Between-group comparisons for ΔECE1, ΔECE2, and ΔECE3 were also performed using the two-sided Mann–Whitney U test with Bonferroni correction across the three Δ parameters. Stratified analyses by IDR morphology and size were conducted descriptively, and between-group differences within each stratum were evaluated using Bonferroni-adjusted Mann–Whitney U tests. Effect sizes for paired analyses were calculated as r = Z/√N, where Z represents the standardized Wilcoxon signed-rank statistic and N denotes the number of paired observations included in the respective analysis. Effect sizes were interpreted according to Cohen’s conventional benchmarks for r (small ≥ 0.10, medium ≥ 0.30, large ≥ 0.50) [15]. The predefined primary outcome parameter was the total incremental cleaning efficacy (ΔECE2). The additional benefit of second-side application (ΔECE3) was considered the mechanistic endpoint. Statistical significance was assumed at a two-sided adjusted *p* ≤ 0.05.

## Results

### Overall experimental interdental cleaning efficacy

**Fig. 4:**
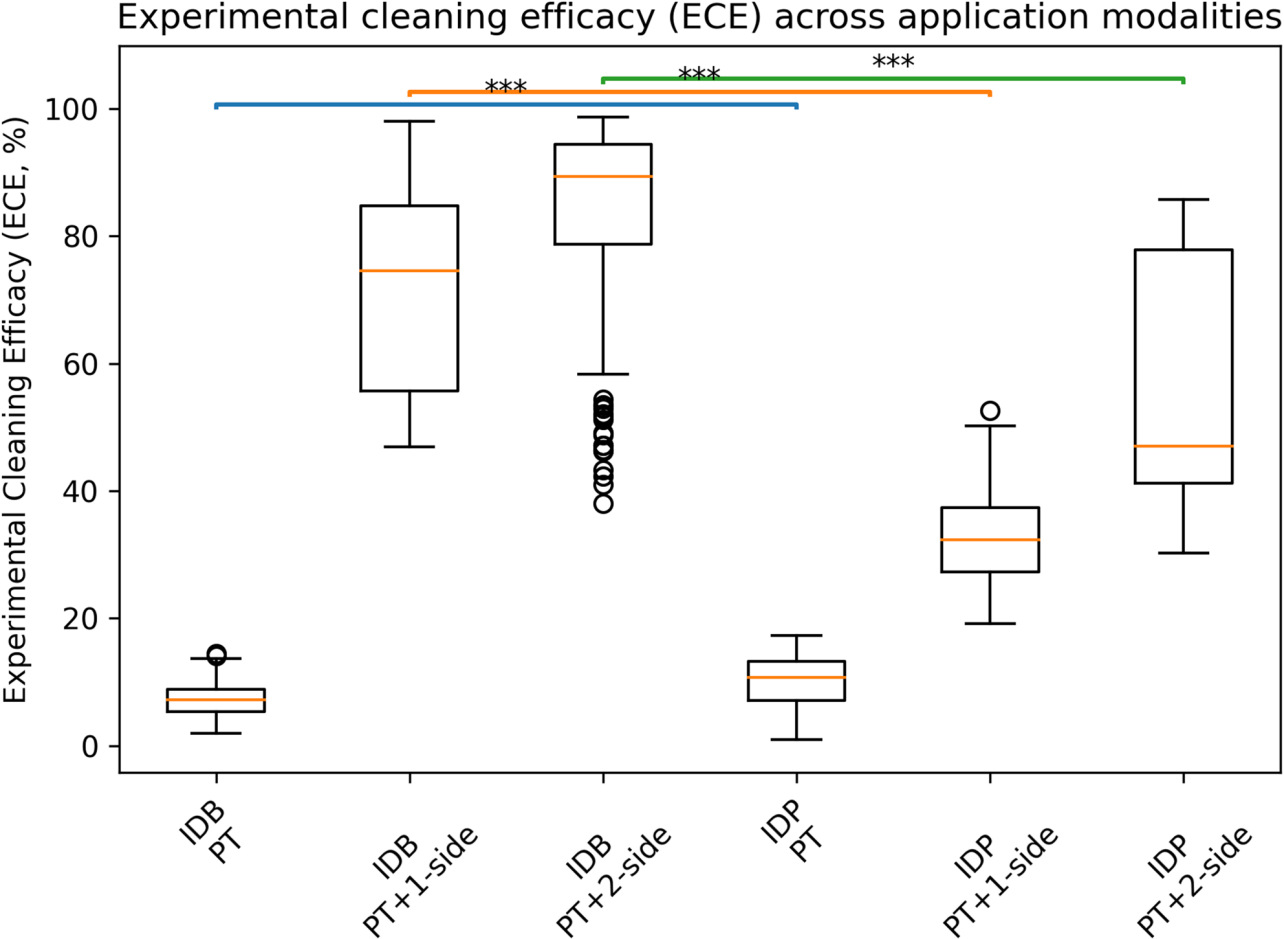
Boxplots illustrate interdental experimental cleaning efficacy (ECE, %) for powered toothbrushing alone (PT), PT followed by one-sided interdental cleaning (PT + one-sided), and PT followed by two-sided interdental cleaning (PT + two-sided). Data are presented separately for interdental brushes (IDB; *n* = 90) and interdental picks (IDP; *n* = 100). Boxes represent the interquartile range (IQR), horizontal lines indicate medians, whiskers denote values within 1.5 × IQR, and circles represent outliers. Between-group comparisons (IDB vs. IDP) were performed using the two-sided Mann–Whitney U test with Bonferroni correction for three comparisons. Adjusted p-values were: PT (*p* < 0.001), PT + one-sided (*p* < 0.001), and PT + two-sided (*p* < 0.001)

ECE following powered toothbrushing alone (PT) was low in both groups. Mean ECE values amounted to 7.50 ± 2.67% for IDB and 10.24 ± 4.05% for IDP, with significantly higher values observed in the IDP group compared with the IDB group (*p* < 0.001). When PT was followed by one-sided interdental cleaning, ECE increased markedly in both groups. Mean values reached approximately 72% for IDB and 33% for IDP (see Table 1), with significantly higher values in the IDB group compared with the IDP group (*p* < 0.001). Following two-sided interdental cleaning, ECE increased further in both groups. Final mean values amounted to approximately 81% for IDB and 55% for IDP, again with significantly higher ECE values in the IDB group compared with the IDP group (*p* < 0.001; Fig. 4).

**Table 1:** Experimental cleaning efficacy (ECE) and incremental benefit (ΔECE) of interdental brushes (IDB; *n* = 90) versus interdental picks (IDP; *n* = 100) in combination with a powered toothbrush (PT)

Outcome	ECE IDB Mean ± SD (IDB Median [IQR])	ECE IDP Mean ± SD (IDP Median [IQR])	IDB vs. IDP *p*-Value
PT	7.48 ± 2.68 (7.26 [5.37–8.86])	10.23 ± 4.07 (10.74 [7.10–13.21])	< 0.001
PT and interdental cleaning (one-sided)	71.68 ± 15.55 (74.60 [55.67–84.75])	33.31 ± 7.77 (32.34 [27.29–37.34])	< 0.001
PT and interdental cleaning (two-sided)	81.19 ± 18.31 (89.34 [78.69–94.39])	54.71 ± 17.63 (47.06 [41.20–77.88])	< 0.001
ΔECE1 incremental benefit (one-sided)	64.19 ± 15.94 (67.29 [48.13–77.55])	23.09 ± 10.85 (20.83 [13.65–32.36])	< 0.001
ΔECE2 incremental benefit (two-sided)	73.70 ± 17.77 (79.41 [70.90–86.42])	44.48 ± 20.61 (33.51 [29.38–71.06])	< 0.001
ΔECE3 additional benefit of second-side application	9.51 ± 12.21 (8.87 [0.53–16.93])	21.40 ± 14.12 (19.88 [9.76–32.72])	< 0.001

**Comparison**	***p*** **-Value**	***p*** **-Value**	
PT vs. PT and interdental cleaning (one-sided)	< 0.001 (*r* = 0.868)	< 0.001 (*r* = 0.868)	
PT alone vs. PT and interdental cleaning (two-sided)	< 0.001 (*r* = 0.868	< 0.001 (*r* = 0.868)	
PT and interdental cleaning (one-sided) vs. PT and interdental cleaning (two-sided)	< 0.001 (*r* = 0.630)	< 0.001 (*r* = 0.858)	

Data are presented as mean ± SD and median [interquartile range (IQR)]. Between-group comparisons (IDB vs. IDP) were performed using the two-sided Mann–Whitney U test

Bonferroni correction for multiple testing was applied across six between-group comparisons. ΔECE1 was calculated as ECE(PT + one-sided) − ECE(PT alone); ΔECE2 as ECE(PT + two-sided) − ECE(PT alone); ΔECE3 as ECE(PT + two-sided) − ECE(PT + one-sided)

Intra-group comparisons were analysed using the paired Wilcoxon signed-rank test. Bonferroni correction was applied separately within each device across three intra-group comparisons. Effect sizes are reported as r = Z/√N

### Incremental cleaning efficacy (ΔECE)

**Fig. 5:**
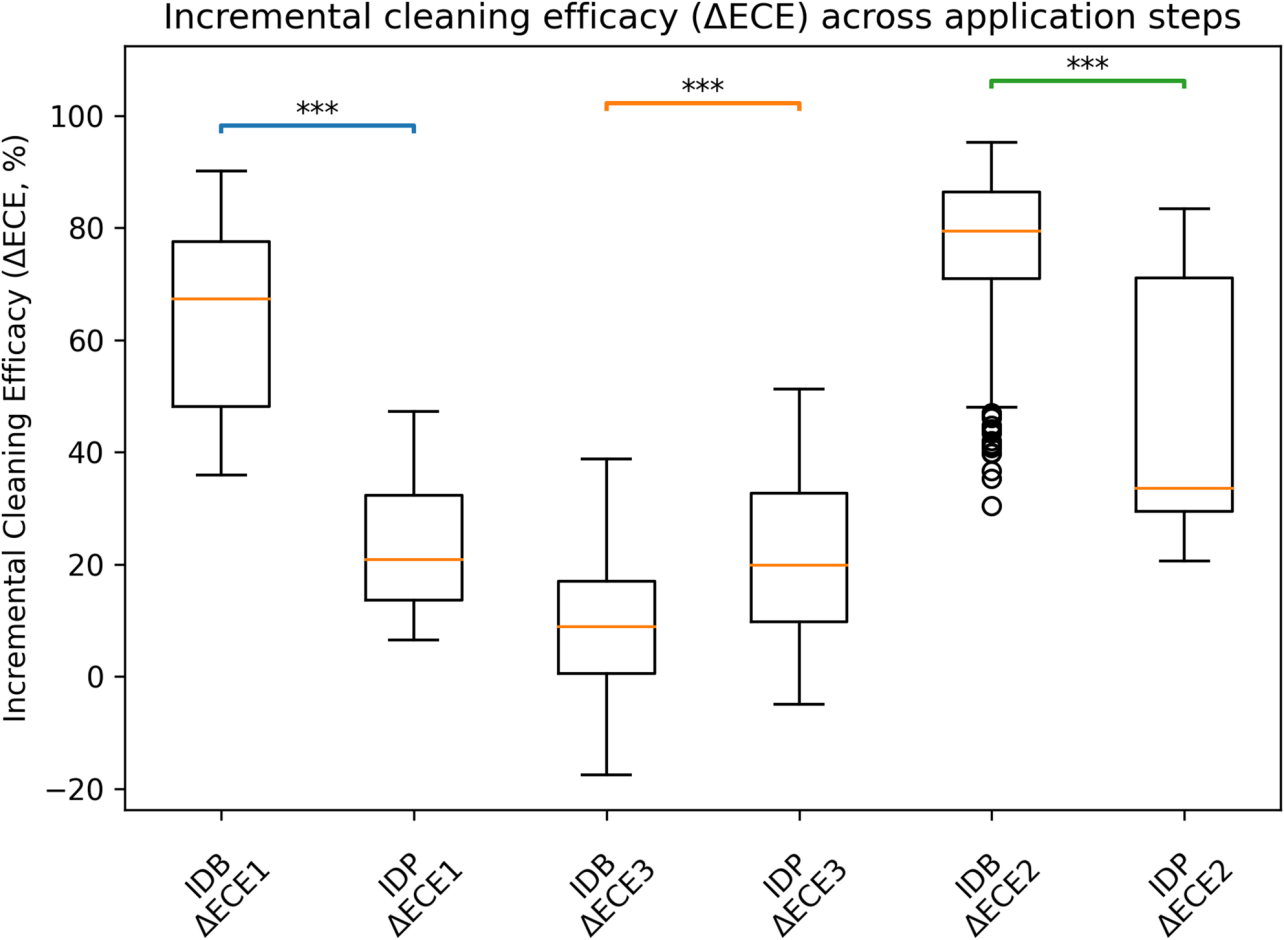
Boxplots illustrate incremental interdental experimental cleaning efficacy (ΔECE, %) in temporal sequence: ΔECE1 (from PT to one-sided), ΔECE3 (from one-sided to two-sided), and ΔECE2 (from PT to two-sided). Data are presented separately for interdental brushes (IDB; *n* = 90) and interdental picks (IDP; *n* = 100). Boxes represent the interquartile range (IQR), horizontal lines indicate medians, whiskers denote values within 1.5 × IQR, and circles represent outliers. Between-group comparisons were performed using the two-sided Mann–Whitney U test with Bonferroni correction for three comparisons. Adjusted *p*-values were significant for all Δ ECE endpoints (ΔECE1 and ΔECE2: IDB > IDP; ΔECE3: IDP > IDB)

The incremental benefit after one-sided application (ΔECE1) was substantially greater for IDB than for IDP (see Table 1; *p* < 0.001; Fig. 5). The total incremental benefit after two-sided application (ΔECE2) likewise remained significantly greater for IDB compared with IDP (*p* < 0.001) and represented the overall superior additive cleaning potential of IDB. Importantly, the additional benefit of the second-side application (ΔECE3) was significantly larger in the IDP group compared with the IDB group (*p* < 0.001; Table 1). Thus, although IDB demonstrated higher absolute and total incremental efficacy, the relative gain attributable specifically to the second-side application was more pronounced for IDP (Fig. 5).

### Within-group comparisons

Within-group analyses demonstrated significant differences between all three application modalities (PT, PT + one-sided, PT + two-sided) for both IDB and IDP (all *p* < 0.001). Effect size analysis revealed consistently large to very large effects (*r* ≈ 0.68–0.87), particularly for the comparison between PT alone and PT + two-sided application in both groups. The total incremental gain after PT (ΔECE2) was 73.7 ± 17.8% for IDB versus 44.5 ± 20.6% for IDP, whereas the second-side incremental gain (ΔECE3) was 9.5 ± 12.2% for IDB and 21.4 ± 14.1% for IDP (Fig. 5; Table 1).

### Stratified analyses by interdental area morphology and size

When stratified by IDR morphology, significant differences between PT alone, PT + IDB, and PT + IDP were observed across all morphological configurations (Table 2). For the isosceles triangle IDR morphology, mean ECE values following two-sided application were 95.29 ± 2.04% for IDB and 80.07 ± 4.38% for IDP. The second-side increment (ΔECE3) was significantly greater for IDP than for IDB (*p* < 0.001), as after one-sided application, ECE amounted to 87.46 ± 6.19% for IDB and 42.24 ± 6.05% for IDP, whereas PT alone yielded 5.69 ± 2.12%. For the concave IDR type, two-sided cleaning potential reached 88.48 ± 3.63% for IDB compared with 41.14 ± 5.56% for IDP. In contrast to the isosceles configuration, the second-side increment was significantly greater for IDB (ΔECE3 difference; *p* = 0.0145). PT alone resulted in 10.64 ± 2.25%. For the convex IDR type, ECE values were lower overall, amounting to 59.79 ± 16.66% for IDB and 47.44 ± 5.14% for IDP following two-sided application. The additional benefit of second-side application was significantly greater for IDP than for IDB (*p* < 0.001).

**Table 2:** Experimental interdental cleaning efficacy (ECE) of powered toothbrushing (PT) and sequential interdental cleaning with interdental brushes (IDB) versus interdental picks (IDP), stratified by IDR size (1.0 mm, 1.1 mm, 1.3 mm) and IDR morphology (isosceles triangle, concave, convex)

	PT alone Mean ± SD (Median [IQR])	PT + IDB (one-sided) Mean ± SD (Median [IQR])	PT + IDP (one-sided) Mean ± SD (Median [IQR])	PT + IDB (two-sided) Mean ± SD (Median [IQR])	PT + IDP (two-sided) Mean ± SD (Median [IQR])
IDR (size 1.0 mm)	9.34 ± 3.47 (8.82 [6.94–10.87])	76.00 ± 18.80 (83.08 [52.56–91.60])	35.21 ± 8.89 (34.70 [27.60–42.11])	76.98 ± 21.55 (90.41 [51.87–94.12])	56.18 ± 18.82 (53.94 [37.12–77.91])
*p*-Value*	One-sided: PT–IDB < 0.001; PT–IDP < 0.001; IDB–IDP < 0.001 Two-sided: PT–IDB < 0.001; PT–IDP < 0.001; IDB–IDP < 0.001				
IDR (size 1.1 mm)	8.56 ± 3.42 (9.62 [5.47–11.50])	63.62 ± 13.64 (55.94 [53.26–78.18])	32.59 ± 7.26 (31.95 [26.57–37.57])	77.74 ± 20.94 (87.69 [53.70–95.48])	51.69 ± 17.77 (43.47 [41.02–54.07])
*p*-Value*	One-sided: PT–IDB < 0.001; PT–IDP < 0.001; IDB–IDP < 0.001 Two-sided: PT–IDB < 0.001; PT–IDP < 0.001; IDB–IDP < 0.001				
IDR (size 1.3 mm)	8.94 ± 4.33 (8.05 [6.00–13.22])	75.41 ± 10.06 (75.55 [66.47–83.76])	32.38 ± 7.11 (29.56 [27.62–35.48])	88.84 ± 5.92 (89.73 [84.92–93.59])	57.26 ± 16.15 (48.62 [45.64–77.81])
*p*-Value*	One-sided: PT–IDB < 0.001; PT–IDP < 0.001; IDB–IDP < 0.001 Two-sided: PT–IDB < 0.001; PT–IDP < 0.001; IDB–IDP < 0.001				
IDR (Isosceles triangle)	5.69 ± 2.12 (6.06 [4.00–7.15])	87.46 ± 6.19 (88.11 [82.66–91.32])	42.24 ± 6.05 (42.88 [37.34–46.93])	95.29 ± 2.04 (95.58 [94.35–96.60])	80.07 ± 4.38 (80.69 [78.37–82.33])
*p*-Value*	One-sided: PT–IDB < 0.001; PT–IDP < 0.001; IDB–IDP < 0.001 Two-sided: PT–IDB < 0.001; PT–IDP < 0.001; IDB–IDP < 0.001				
IDR (Concave)	10.64 ± 2.25 (10.74 [9.24–12.13])	70.90 ± 13.21 (74.60 [56.27–80.76])	32.08 ± 3.88 (32.34 [28.80–35.25])	88.48 ± 3.63 (89.34 [87.41–90.50])	41.14 ± 5.56 (40.81 [36.96–44.73])
*p*-Value*	One-sided: PT–IDB < 0.001; PT–IDP < 0.001; IDB–IDP < 0.001 Two-sided: PT–IDB < 0.001; PT–IDP < 0.001; IDB–IDP < 0.001				
IDR (Convex)	10.16 ± 4.31 (10.19 [6.23–14.37])	56.67 ± 6.13 (55.26 [51.60–61.91])	26.02 ± 3.11 (25.82 [24.06–27.90])	59.79 ± 16.66 (52.53 [47.13–78.29])	47.44 ± 5.14 (46.13 [43.71–51.19])
*p*-Value*	One-sided: PT–IDB < 0.001; PT–IDP < 0.001; IDB–IDP < 0.001 Two-sided: PT–IDB < 0.001; PT–IDP < 0.001; IDB–IDP 0.019				

Data are presented as mean ± SD and median [interquartile range (IQR)]. PT-alone values were pooled within each stratum. *Pairwise between-group comparisons were performed using two-sided Mann–Whitney U tests. Bonferroni correction was applied within each stratum across six comparisons (three one-sided and three two-sided comparisons)

Similarly, stratification by IDR size demonstrated size-dependent differences in ΔECE3. For small interdental areas (1.0 mm), the second-side increment was significantly greater for IDP compared with IDB (*p* < 0.001). For IDR of 1.1 mm size, no statistically significant difference in ΔECE3 between IDB and IDP was observed (*p* = 0.727). For IDR of 1.3 mm size, the second-side increment was again significantly greater for IDP (*p* < 0.001).

Across all IDR morphologies and sizes, PT alone consistently demonstrated the lowest interdental cleaning efficacy (Table 2). While IDB achieved higher absolute and total cleaning potential across strata, the additional benefit attributable specifically to bilateral application (ΔECE3) was morphology- and size-dependent. ΔECE3 was significantly greater for IDP than for IDB in isosceles triangle (*p* < 0.001) and convex morphologies (*p* < 0.001), as well as in small (1.0 mm, *p* < 0.001) and large IDRs (1.3 mm, *p* < 0.001). In contrast, for the concave morphology, the second-side increment was significantly greater for IDB (*p* = 0.0145), and no significant difference was observed for 1.1 mm IDRs (*p* = 0.727; Table 2).

## Discussion

The present in vitro study was designed to evaluate the additional cleaning potential achieved by bilateral interdental application following powered toothbrushing and to compare the performance of conventional IDBs and IDPs. The findings confirm that additional bilateral interdental cleaning significantly increased ECE compared with PT alone. However, our hypothesis that IDPs would exhibit a greater bilateral incremental benefit than IDBs was only partially supported. The magnitude of the total additional cleaning potential was substantially greater for IDB, with a mean ΔECE2 of approximately 74%, compared with 45% for IDP, corresponding to an absolute difference of nearly 30%. In contrast, the additional benefit attributable specifically to second-side application (ΔECE3) was greater for IDP (approximately 21%) than for IDB (approximately 10%). The low interdental cleaning potential observed after PT alone is consistent with previous experimental observations demonstrating the limited penetration of toothbrush bristles into approximal areas [16]. In the present in vitro model, powered toothbrushing alone resulted in cleaning of less than 10% of the simulated interdental surface.

Given that the IDR replicas had a depth of 10 mm, this proportion would correspond roughly to an effective penetration of approximately 1 mm into the interdental space. Although this calculation is illustrative rather than a direct measurement of penetration depth, it highlights the limited mechanical access of toothbrush bristles to approximal surfaces. Representative images of the experimental setup demonstrate that cleaning after PT alone was largely restricted to the coronal entrance of the interdental space (see Supplementary Figure S1). This observation is consistent with clinical evidence demonstrating that toothbrushing alone is insufficient for effective interdental plaque removal and that adjunctive interdental cleaning is required to reduce plaque and gingival inflammation [17, 18].

The superiority of IDBs observed in the present study aligns with systematic reviews and consensus reports indicating that IDBs are generally more effective than floss or elastomeric picks in reducing plaque and gingival inflammation, particularly in open interdental spaces [4, 19, 20]. The recent patient-centred interdental cleaning consensus also emphasizes the preferential recommendation of interdental brushes when anatomy allows their use [1].

However, most available clinical studies report overall plaque or bleeding outcomes and do not quantify the incremental benefit achieved by sequential bilateral interdental application following powered toothbrushing under standardized conditions. The present study extends existing knowledge by specifically isolating and quantifying both the total incremental effect after PT (ΔECE2) and the additional benefit of second-side application (ΔECE3). This controlled approach enables a direct comparison of device performance independent of patient-related variability. The greater additional cleaning effect observed for IDBs may be explained by their cylindrical bristle configuration, which allows circumferential contact with proximal surfaces [3] and improved adaptation to the interdental space. This configuration likely enhances direct mechanical contact with the tooth surfaces, thereby improving biofilm disruption [18, 21]. In contrast, the more pronounced second-side incremental gain observed for IDPs suggests that their conical elastomeric design may benefit disproportionately from bilateral access, possibly due to progressive engagement of the tapered tip within the interdental space when approached from opposing directions. IDPs are often perceived as easier to handle and more comfortable for patients [22]. However, in our previous in vitro investigation under dry conditions [3], IDPs required significantly higher cleaning forces while achieving substantially lower cleaning efficacy compared with conventional IDBs. In a subsequent study including artificial saliva [23], these forces were markedly reduced, suggesting a lubrication-dependent effect. Nevertheless, even under moist conditions, cleaning efficacy remained lower for IDPs, indicating that perceived ease of use may not necessarily correspond to superior mechanical cleaning performance. It should be noted that perceived ease of use has been associated with higher patient-reported compliance in clinical studies [22]. However, improved adherence with a less mechanically effective device does not necessarily result in superior clinical outcomes compared with less frequent use of a more effective device. The extent to which in vivo compliance differences may offset the mechanical performance gap between IDB and IDP remains to be investigated in appropriately powered randomised clinical trials.

The influence of interdental morphology observed in the present study further supports the importance of anatomical adaptation. Previous work has demonstrated that interdental morphology significantly affects the performance of mechanical cleaning devices [6]. Although IDBs achieved higher absolute and total incremental cleaning efficacy across IDR morphologies and sizes, the relative bilateral gain (ΔECE3) varied depending on anatomical configuration. For example, combined ECE reached approximately 95% in isosceles triangle morphologies and 88% in concave configurations for IDBs, compared with substantially lower values for IDPs. These findings are in line with our previous experimental investigation [3], in which IDBs achieved cleaning efficacy of approximately 58% compared with 18% for IDPs across morphologies.

### Clinical implications

Current clinical guidelines recommend interdental cleaning as an integral component of daily oral hygiene, particularly in patients with open interdental spaces [4]. The present data reinforce these recommendations by quantitatively demonstrating the limited efficacy of toothbrushing alone and the substantial additional benefit of interdental cleaning. Bilateral application further enhanced cleaning performance beyond one-sided use. While patient-centred factors such as access, dexterity, and preference must guide device selection [1], the mechanical cleaning performance of IDBs in terms of total incremental efficacy appears superior under standardized conditions. At the same time, IDPs demonstrated a comparatively greater relative gain from bilateral application, suggesting that their effectiveness may benefit particularly from buccal and oral access. However, clinical effectiveness depends not only on mechanical performance but also on patient adherence, accessibility, and correct device sizing. Based on the present data, bilateral (buccal and oral) interdental application should be considered the preferred technique whenever anatomically feasible, as it consistently provided higher cleaning efficacy than one-sided application for both device types. Clinicians and dental hygienists are encouraged to instruct patients accordingly, with realistic expectations regarding the additional benefit, which amounted to approximately 10% for IDB and approximately 21% for IDP. Patient-individual factors such as dexterity, access, and device preference should continue to guide final device selection [1].

### Limitations

Several limitations of the present in vitro model must be acknowledged. Although our validated experimental set-up allows standardized and reproducible quantification of ECE [3, 6], the results cannot be directly extrapolated to clinical conditions. Interdental devices were introduced in a strictly linear direction to ensure reproducibility. However, this standardized insertion path does not reflect potential variations in angulation, pressure, and access occurring under real-life intraoral conditions. Furthermore, the artificial biofilm model does not fully replicate the structural complexity and adhesion characteristics of mature in-vivo plaque. The Occlu Spray Plus served as a standardised plaque surrogate that enables reproducible quantification of cleaned surface areas. However, as discussed in detail in our previous validations of this experimental setup [6], the surrogate differs from mature in vivo interdental biofilm in its adhesion strength, structural heterogeneity, and biomechanical properties, and does not replicate the pathogenic characteristics of genuine periodontal biofilm, including its microbial composition and capacity to induce inflammatory responses. The clinical relevance of in vitro cleaning potential must therefore ultimately be assessed in terms of clinically meaningful outcomes such as plaque reduction, bleeding on probing, and periodontal pocket depth – endpoints that cannot be modelled in the present in vitro framework (cf. [6, 24]). The absence of saliva flow dynamics, patient-related factors (e.g., dexterity, compliance), and biological variability represents additional limitations [23]. Device selection and performance in clinical practice may therefore differ from standardized laboratory conditions. In the present study, device sizes were selected according to predefined IDR dimensions using standardized fitting criteria based on our previously validated experimental model [3], ensuring a structured and non-subjective size-matching procedure. Consequently, no separate statistical analysis of individual product sizes was performed. While this approach reflects clinically appropriate device selection, it does not allow conclusions regarding size-specific performance within each device category.

Although PT values were obtained using the same sequential experimental protocol, IDB and IDP series were conducted on separate replica sets and baseline differences between series were observed across IDR morphologies and sizes (Table 2). As measurements were conducted on separate replica sets, small systematic variations related to biofilm coating characteristics, device positioning or alignment, or brushing-head condition over time cannot be entirely excluded [6]. However, the primary outcome parameter (total incremental cleaning efficacy, ΔECE2), which accounts for baseline values by evaluating sequential change within each specimen, was not materially affected by these differences.

Future studies should investigate whether the quantitative cleaning advantage of bilateral application observed in vitro translates into measurable clinical benefits, including reductions in plaque index scores, bleeding on probing, and periodontal pocket depth. Additionally, qualitative analyses of interdental biofilm composition following different application strategies, as well as the influence of factors such as food composition, saliva flow, and nutritional habits on in vivo cleaning effectiveness, would advance our understanding of optimal interdental hygiene behaviour. Despite these limitations, and given the lack of a precise and reproducible quantitative method for measuring interdental plaque removal in vivo, the present model provides a controlled framework for comparative assessment of interdental cleaning performance across defined interdental morphologies and sizes.

## Conclusions

Within the limitations of this in vitro study, powered toothbrushing alone was insufficient for effective interdental cleaning, and bilateral device application substantially improved cleaning efficacy beyond one-sided use. Cylindric IDBs demonstrated superior overall performance across all tested conditions, while conical IDPs derived a comparatively greater relative benefit from bilateral access. These findings support bilateral application as the preferred technique and highlight the need for future in vivo studies to confirm whether the observed cleaning benefits translate into meaningful reductions in interdental plaque and gingival inflammation.

## Supplementary Information

Supplementary Material 1: Supplemental Figure S1. Representative images of a convex interdental region (IDR), size 1.0, illustrating the experimental sequence and corresponding digital plaque quantification. (a) Interdental cleaning performed with an interdental brush (IDB; ISO size 1, metal core) versus (b) with a metal-free elastomeric interdental pick (IDP; size S). For each panel (a, b), the four images on the left depict the sequential experimental conditions: baseline (stained surface), after powered toothbrushing (PT) on both sides, after PT plus unilateral interdental cleaning, and after PT plus bilateral interdental cleaning. The corresponding images on the right show the respective digital analysis masks used for quantification of exposed coloured area (ECE) at each stage (baseline; after PT bilaterally; after PT + unilateral interdental cleaning; after PT + bilateral interdental cleaning). Red areas in the threshold-based differential image indicate regions with significant changes in the stained surface, i.e., areas removed by cleaning that were subsequently quantified for analysis. This figure exemplifies the standardized image-based workflow applied for calculation of ΔECE across experimental conditions.

## Data Availability

The datasets used and/or analysed during the current study are available from the corresponding author on reasonable request.

## Abbreviations

ECE: Experimental cleaning efficacy
IDB: Interdental brushes
IDP: Interdental picks
IDR: Interdental area
PT: Powered toothbrush
