# Supplementary figures and images for: Bilateral interdental cleaning after powered toothbrushing: an in vitro comparison of interdental brushes and elastomeric interdental picks
Source: BMC Oral Health. 2026 Jun 18;26:1071. doi: 10.1186/s12903-026-08946-8 (PMC13277008; doi:10.1186/s12903-026-08946-8)

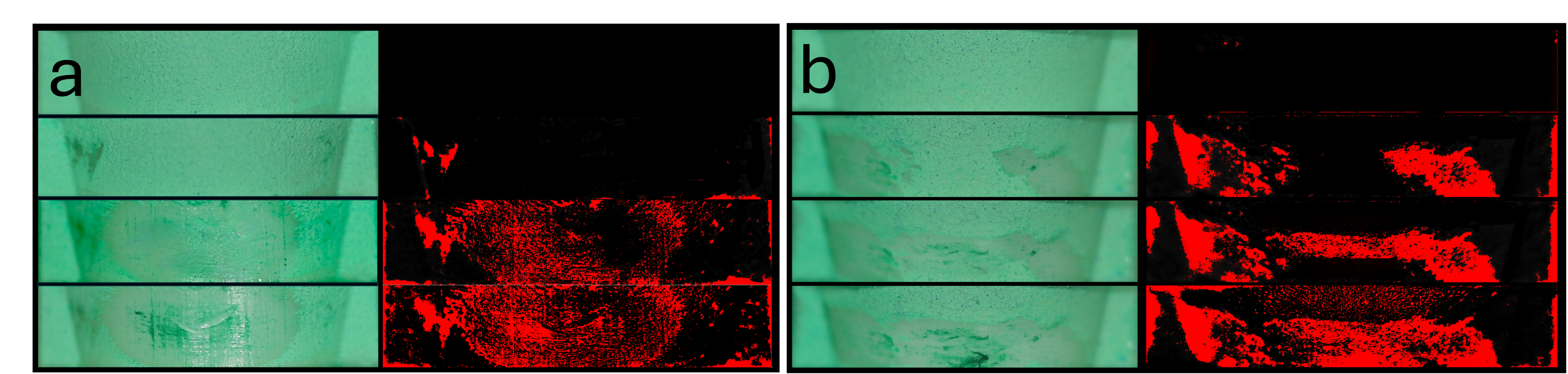

Supplement: Supplementary file 1 — Supplementary Material 1: Supplemental Figure S1. Representative images of a convex interdental region (IDR), size 1.0, illustrating the experimental sequence and corresponding digital plaque quantification. (a) Interdental cleaning performed with an interdental brush (IDB; ISO size 1, metal core) versus (b) with a metal-free elastomeric interdental pick (IDP; size S). For each panel (a, b), the four images on the left depict the sequential experimental conditions: baseline (stained surface), after powered toothbrushing (PT) on both sides, after PT plus unilateral interdental cleaning, and after PT plus bilateral interdental cleaning. The corresponding images on the right show the respective digital analysis masks used for quantification of exposed coloured area (ECE) at each stage (baseline; after PT bilaterally; after PT + unilateral interdental cleaning; after PT + bilateral interdental cleaning). Red areas in the threshold-based differential image indicate regions with significant changes in the stained surface, i.e., areas removed by cleaning that were subsequently quantified for analysis. This figure exemplifies the standardized image-based workflow applied for calculation of ΔECE across experimental conditions. [file 12903_2026_8946_MOESM1_ESM.png]
